# A SIX1 Homolog in *Fusarium oxysporum* f. sp. *conglutinans* Is Required for Full Virulence on Cabbage

**DOI:** 10.1371/journal.pone.0152273

**Published:** 2016-03-24

**Authors:** Erfeng Li, Gang Wang, Jiling Xiao, Jian Ling, Yuhong Yang, Bingyan Xie

**Affiliations:** 1 Institute of Vegetables and Flowers, Chinese Academy of Agricultural Sciences, Beijing 100081, China; 2 Hu'nan Watermelon and Muskmelon Institute, Changsha 410125, China; Soonchunhyang University, REPUBLIC OF KOREA

## Abstract

*Fusarium oxysporum* is a soil-born fungus that induces wilt and root rot on a variety of plants. *F*. *oxysporum* f. sp. *conglutinans* (Foc) can cause wilt disease on cabbage. This study showed that a homolog of SIX1 protein in the *Arabidopsis* infecting isolate Fo5176 (Fo5176-SIX1) had four isoforms in the conidia of Foc by proteomic analysis. Thus, we analyzed the roles of protein Foc-SIX1. Gene expression analysis showed that, compared to the expression in mycelia, dramatically altered expression of *Foc-SIX1* could be detected after infecting cabbages, and *Foc-SIX1* was highly expressed in conidia under axenic culture condition. Furthermore, we knocked out the *Foc-SIX1* gene and found that Foc-ΔSIX1 mutants had significantly reduced virulence compared with wild type isolate, and full virulence was restored by complementation of Foc-ΔSIX1 mutants with *Foc-SIX1*. Thus, we concluded that SIX1 in Foc was required for full virulence on cabbage. We also complemented Foc-ΔSIX1 with *SIX1* gene in *F*. *oxysporum* f. sp. *lycopersici* (Fol) and found Foc-ΔSIX1::Fol-SIX1 mutants did not affect the virulence of Foc-ΔSIX1. The results confirmed that Fol-SIX1 was not capable of replacing the role of Foc-SIX1 in Foc on the disease symptom development of cabbage. The roles of Fol-SIX1 on virulence might rely on host specificity.

## Introduction

The soil-born fungus *Fusarium oxysporum* f. sp. *conglutinans* (Foc) causes vascular wilt disease of cabbage. *Fusarium* wilt of cabbage originated in the United States and was first reported in China in 2001 [1].

The interaction between *F*. *oxysporum* f. sp. *lycopersici* (Fol) and tomato has become a model system for studying the effectors of the pathogen and R proteins from the host [2]. The secreted-in-xylem (SIX) proteins were first reported in the xylem sap of Fol-infected tomato [3]. In total, eleven SIX proteins have been identified in Fol so far, and the functions of most of these proteins remain unknown [2]. Three of these proteins (SIX1/SIX3/SIX4) could interact with the tomato genes (I/I-1/I-2/I-3) to induce resistance [4–6]. Genomic analysis demonstrated that most of *SIX* genes were primarily located on supernumerary pathogenicity chromosomes that could transfer between strains [7]. The small and cysteine-rich SIX1 effector protein was secreted by Fol during colonization of tomato xylem vessels and was required for virulence in a fully susceptible tomato genotype [8]. SIX1 could interact with I-3, which the tomato plants carry to trigger a resistant recognition reaction [6]. *SIX1* was strongly up-regulated during colonization of the host plant [9], and many effector genes were unexpressed or weakly expressed in axenic cultures and up-regulated during infection [10]. Moreover, it was reported that the SIX4 homolog Fo5176-SIX4, with >99% amino acid identity to Fol-SIX4, was required for full virulence in the *Arabidopsis* infecting isolate Fo5176 [11]. Despite the extensive researches performed in Fol, little research has been conducted on the possible role in pathogenicity of *SIX* genes outside Fol.

In the present study we identified four interesting proteins in the conidia of Foc as Fo5176-SIX1 (gi|342888423) during proteomic analysis. Fo5176-SIX1, identified in the isolate Fo5176, shared 74% identity to Fol-SIX1, which was identified as the avirulence protein of I-3-mediated resistance in tomato [5,6,11]. Compared to *SIX1* expression in mycelia, *SIX1* expression was dramatically altered after Foc infected cabbages. In this work, we also found that *Foc-SIX1* expression was also high in conidia under axenic culture condition. Thus, the objective of this study was to explore the roles played by SIX1 in Foc infection of cabbage.

## Methods

### Fungal strains, plant materials and culture conditions

Cabbage infecting Foc wild-type isolate (52557^-TM^) was obtained from the Beijing Academy of Agriculture and Forestry Sciences and selected for use in this study. Foc wild type isolate was routinely grown on potato dextrose agar (PDA) at 28°C. Deletion mutants (Foc-ΔSIX1: D1 and D2) and complementation mutants (Foc-ΔSIX1::Foc-SIX1: C1, C2, C3 and C4; Foc-ΔSIX1::Fol-SIX1: T1 and T2) were maintained on PDA containing 100 μg/mL hygromycin B or 100 μg/mL neomycin under the same conditions.

A Foc-susceptible cabbage cultivar “Zhong gan 21” was used in this study [12]. Seedlings were grown in plastic pots filled with an autoclaved mixture of vermiculite and turf (1:1, v/v) in a glasshouse at approx. 25°C and a 16-h light photoperiod until the 2–3 true leaf stage.

### Protein extraction, quantification and 2-DE

Total proteins were extracted from the conidia of Foc by the borax/PVPP/Phe (BPP) protocol [13]. This method combined ammonium sulfate saturated-methanol and BPP to isolate proteins. Protein concentrations were quantified by the Bradford method with bovine serum albumin (BSA) as the standard [14]. Then, 24 cm, pH 4–7 linear gradient IPG strips were rehydrated, and IEF was carried out using the Hoffer IEF100 at 20°C for focusing. After equilibration, the strips were transferred to 12.5% SDS-PAGE to separate proteins in the second dimension (Ettan DALT six, GE Healthcare) [15]. Three biological replicates were performed to ensure protein reproducibility.

### Gel staining and image analysis

The gels were visualized by the GAP method, which used CBB-G250, ammonium sulfate, and phosphoric, and scanned. These images were analyzed with Image Master 2D Platinum Software (Version 5.0, GE Healthcare).

### MS identification and database search

Proteins were identified by MALDI TOF/TOF MS/MS (Bruker Daltonics, Bremen, Germany) and analyzed with flexAnalysis software (Version 3.2) [16]. Spectra were then analyzed using a MASCOT search against the taxonomy of fungi in the non-redundant NCBI (NCBInr) database. The MS/MS ion search parameters were conducted as MS/MS tolerance of ±0.5 Da with allowances for oxidation (M) modifications and for one missed cleavage as well as with fixed modification of cysteine by carboxymethyl (Carbamidomethylation, C). The optimal match was considered according to a higher Mascot score. Proteins with protein score confidence intervals above 95% were considered as confident identifications. Furthermore, we used a BLASTP search to find SIX1 homologs. The protein alignment was performed and the tree was generated by the neighbor-joining method using MEGA 6 with 1000 bootstraps based on alignments of full amino acid sequences of homologs.

### Mycelia, conidia, infected root collection and RNA extraction

The isolate was cultured in autoclaved potato dextrose broth (PDB) at 28°C on a shaker at 150 rpm for 48 h. Mycelia were then filtered through four layers of sterilized lens paper and washed with sterile distilled water. The conidia were separated from the conidial suspensions by centrifugation at 5000 g for 15 min at room temperature. The conidia were obtained for virulence studies and protein and RNA extraction. The mycelia were used for RNA extraction. After 7 days post-inoculation (dpi), the infected cabbage seedling roots were collected. For each sample, three independent biological replicates were conducted. RNA extraction, cDNA synthesis and qRT-PCR reactions were performed as described previously [17].

### Quantification of *SIX1* gene expression using qRT-PCR

Gene sequences were obtained from the published Fo5176 reference genome sequence and Foc genome sequence [7]. To compare *SIX1* expression levels in mycelia, conidia and *in planta* conditions, two housekeeping genes, elongation factor 1-α (*EF-1α*) and *β-tubulin*, were used for normalization and calculation of the relative expression values using the 2^-ΔΔCt^ method [9,18]. The *β-tubulin* gene primers were designed from FOXG_06228 in Fol and had been previously verified for use in Fo5176 [11]. The qRT-PCR reactions for each gene were repeated four times. All primers were tested for expected size and specific for the predicted PCR product and were listed in S1 Table.

### Deletion and complementation of *SIX1* in *F*. *oxysporum* f. sp. *conglutinans*

Deletion of the *SIX1* gene was generated using the overlap PCR and split marker gene strategy. The strategy used a DNA mixture containing overlapping truncations of the selectable marker to enable homologous integration during transformation [19–22]. The target gene was replaced with the 1.4 kb fragment that encoded hygromycin B phosphotransferase as a selectable marker. A 1165 bp region upstream from the start codon and a 1211 bp region downstream from the stop codon of the target gene were amplified from genomic DNA of Foc with primers 1-F/(2+5)-R and (8+3)-F/4-R. Marker tail sites were introduced 3’ of the upstream fragment and 5’ of the downstream fragment. The split replacement gene was amplified from vector PCH-SGFP carrying the *hph* gene [23]. The two split marker transformation components were amplified by two rounds of PCR. These steps are shown in the S1 Fig. The PCR mixtures of the two split marker transformation components were directly used for protoplast-mediated transformation [24]. The correct deletion mutants (Foc-ΔSIX1) were confirmed by PCR and Southern hybridization. Genomic DNA was digested with *Sal* I, separated in a 0.8% agarose gel, and hybridized with a 308 bp probe. All primers related to gene deletion and deletion mutant confirmation were listed in S2 and S3 Tables.

Complementation of Foc-ΔSIX1 was achieved by transformation with approximately 3.0 kb of *Foc-SIX1* spanning from -1838 bp (promoter relative to start codon) to 1132 bp (ORF and 3’ UTR). The resulting amplicon was cloned into *Bam*HI/*Eco*RI-digested KN to produce Foc-SIX1-comp-KN. This construct was transformed into Foc-ΔSIX1 to produce Foc-ΔSIX1::Foc-SIX1 by protoplast-mediated transformation. The transformants were selected on PDA with neomycin and confirmed by PCR amplification. All primers related to gene complementation and transformants confirmation are listed in S4 Table.

### Expression of Fol-SIX1, the Foc-SIX1 homolog in *F*. *oxysporum* f. sp. *lycopersici*, in the deletion mutants Foc-ΔSIX1

To characterize the role of the homolog Fol-SIX1 in Foc, the 1.9 kb of *Fol-SIX1* spanning from -832 bp to 1091 bp off Fol genomic DNA was amplified and cloned into *Bam*HI/*Eco*RI-digested KN to produce Fol-SIX1-com-KN. This construct was transformed into Foc-ΔSIX1 to produce Foc-ΔSIX1::Fol-SIX1 strain by protoplast-mediated transformation. The transformants were selected on PDA with neomycin and confirmed by PCR amplification. All primers related to gene complementation and transformants confirmation are listed in S5 Table.

### Transformation procedures mediated by PEG-CaCl2

Protoplasts were prepared as described previously, and their concentration was adjusted to 1×10^8^ protoplasts/mL [25]. The protoplasts were then transferred into Eppendorf tubes at 100 μL/tube and kept at -80°C for future use. Transformation components were added to 100 μL of frozen protoplast suspension and mixed gently with a pipette tip. Next, 160 μL of PEG solution was dropped into the DNA-protoplasts suspension. The protoplast suspension was then mixed with molten regeneration medium (minimal medium) and poured onto regeneration medium. Deletion mutants and complementation transformants were selected and maintained on PDA medium containing 100 μg/mL hygromycin B or 100 μg/mL neomycin.

### Inoculum preparation and inoculation procedure

Conidial suspension of Foc and their mutants were used as inocula, which was described above. Inoculum concentration was adjusted to 1×10^6^ conidia/mL and the root-dip method was used in this study [26]. Cabbage seedlings dipped in sterilized distilled water served as non-inoculated controls. For each isolate, three independent biological replicates were carried out and a total of more than thirty seedlings were inoculated in every replicate.

### Disease assessment and statistical analysis

Disease symptoms were assessed according to the disease severity, divided into five levels according to pre-established rating scales [12,26]. The scoring standard of cabbage susceptibility to Foc was evaluated by statistical analysis: index = 0–5, where “0” meant no evidence of disease symptoms; “1” represented only one leaf showed slight or moderate yellowing; “2” indicated that two leaves showing slight or moderate yellowing; “3” meant all leaves showed moderate yellowing or wilt except for interior leaves; “4” meant all leaves showed severe yellowing or wilt; and “5” indicated that the seedling was dead. The disease index (DI) was calculated according to the rating scales [12]. The statistical analysis of differences in DI was performed by two-way ANOVA procedures using Duncan’s multiple range test in SAS software. The statistical results are shown as the mean ±SE (standard error).

## Results and Discussion

### A conserved SIX1 homolog was identified in Foc by proteomic analysis

**Fig 1:**
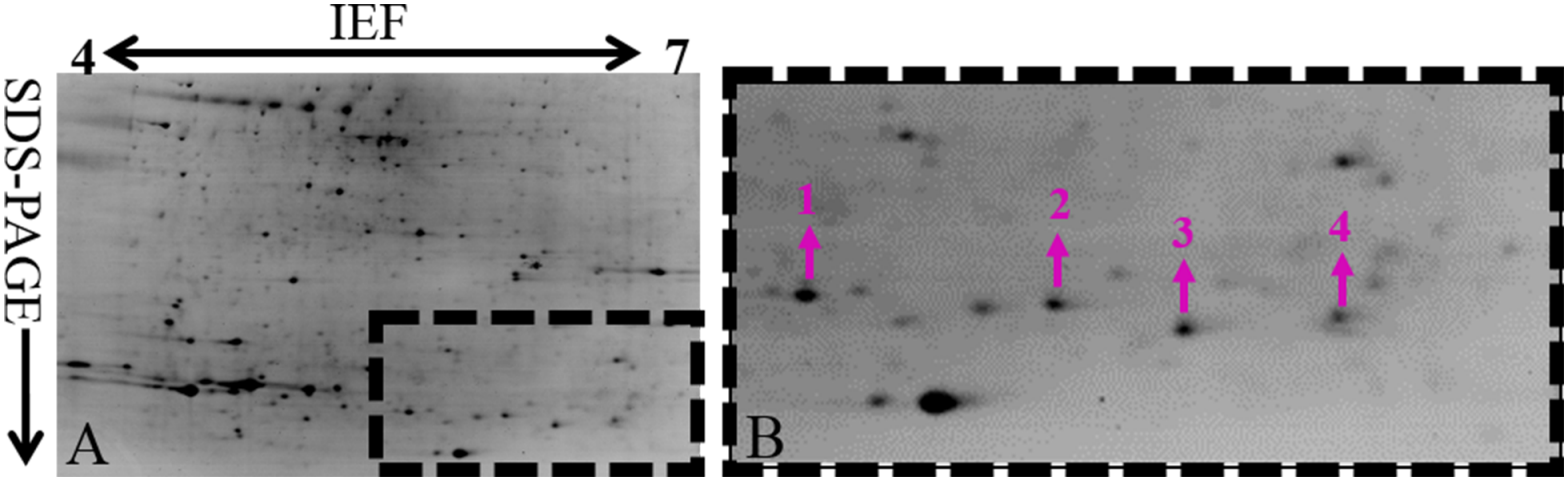
Reference maps from 2-DE analysis of conidial proteins from *F*. *oxysporum* f. sp *conglutinans*. Proteins were separated by using 24 cm, pH 4–7 IPG strips for IEF and followed by 12.5% SDS-PAGE. The gels were stained by GAP method, which used CBB-G250, ammonium sulfate, and phosphoric acid. A: Gel of conidia proteins from Foc grown in PDB. B: The four proteins (spots 1, 2, 3 and 4), named Foc-SIX1 with high abundance in Foc, were indicated by arrows and numbers and listed in Table 1; Figure B is the partial enlarged details of the rectangular box in Figure A.

**Fig 2:**
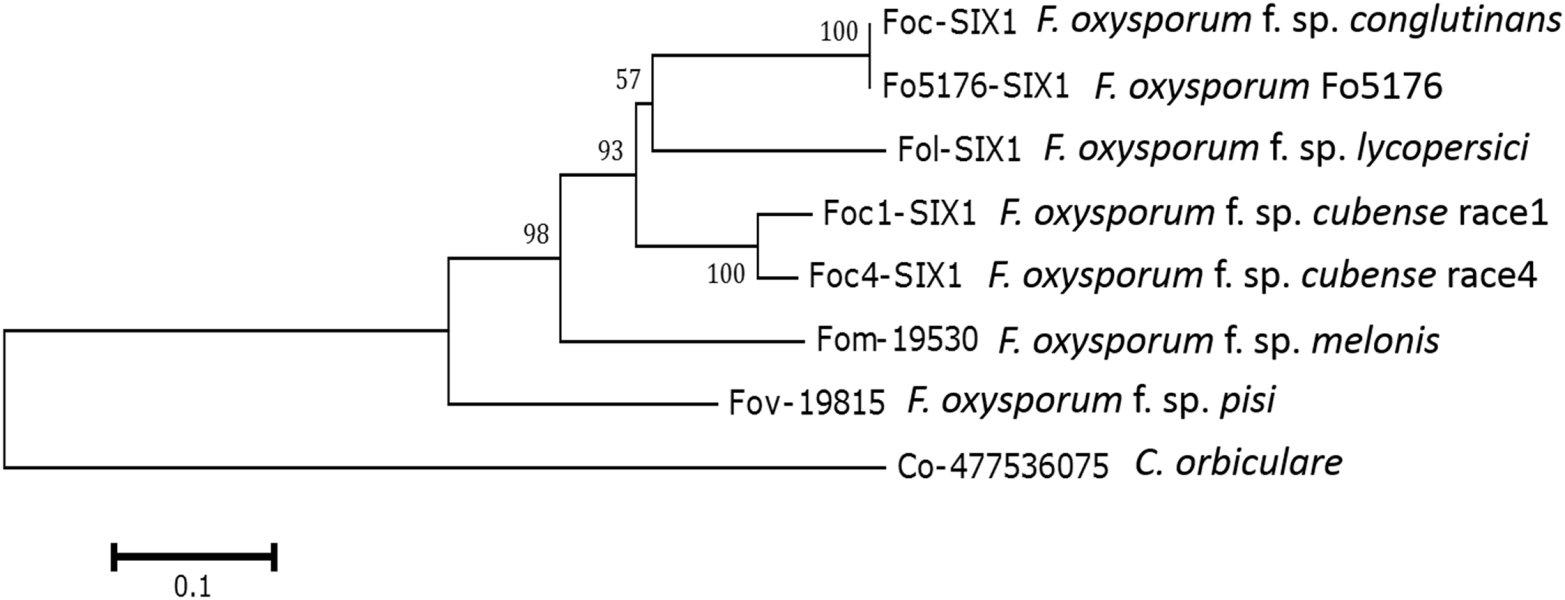
Phylogenetic analysis of Foc-SIX1 by using neighbor-joining method. The tree was generated based on alignments of full amino acid sequences of homologs using MEGA 6 with 1000 bootstraps.

Two-dimensional electrophoresis (2-DE) and mass spectrometry (MS), combined with careful systematic and statistical analysis, were used to reliably separate and identify pathogenesis-related proteins in Foc. In total, 461 protein spots were detected in the conidia of Foc. Among these, 20 protein spots were identified. From the identification results, it was noteworthy that four protein spots (1, 2, 3 and 4) had higher identities with Fo5176-SIX1 (Fig 1A and 1B). The Fo5176 genomic sequence was previously obtained using Illumina and 454 sequencing [7,11]. These four proteins, which were nicely separated on the gels, were located in horizontal strings with identical functional annotation. We have sequenced the genome of Foc. Only one homolog of Fo5176-SIX1 was found in Foc genome using BLASTN with E-value cutoff of 1e-5. The gene *Foc-SIX1* had 100% identity with *Fo5176-SIX1*. This result indicated that a single gene product with post-translational modifications changing the *p*I resulted in four isoforms of this protein in Foc (Fig 1B). We named this protein Foc-SIX1. These four identified protein isoforms were listed in Table 1 (See S6 Table, S1 File). BLASTN searches did not reveal the identification of this protein with known functions, but uncovered its homologs in other *F*. *oxysporum forma speciales* and *Colletotrichum orbiculare*. Phylogenetic analysis showed that Foc-SIX1 was more similar to Fo5176-SIX1 and Fol-SIX1 proteins than to SIX1 of *F*. *oxysporum* f. sp. *cubense* (Fig 2). Foc-SIX1 shared 100% and 74% identity with Fo5176-SIX1 and Fol-SIX1, respectively. These two identified proteins were annotated as small, cysteine-rich proteins and contributed to virulence of the pathogens. Therefore, we have focused on Foc-SIX1 in this study.

**Table 1:** Identification of the four proteins indicated in Fig 1 from the conidia of Foc.

Spot No.^a^	Acc. ID^b^	Protein identity	Organism	Theor. *P*I/Mr^c^	Exper. *P*I/Mr^d^	MP^e^	SC%^f^	Loc^g^	MS^h^
**1**	342888423	Fo5176-SIX1	*F*. *oxysporum*	8.54/31.3	6.38/16.8	1	5	S	86
**2**	342888423	Fo5176-SIX1	*F*. *oxysporum*	8.54/31.3	6.87/16.9	1	5	S	58
**3**	342888423	Fo5176-SIX1	*F*. *oxysporum*	8.54/31.3	7.01/15.9	2	10	S	182
**4**	342888423	Fo5176-SIX1	*F*. *oxysporum*	8.54/31.3	7.18/15.8	1	5	S	65

^a^Designated spot number as indicated and marked on the 2-DE gel in Fig 1;

^b^The accession the accession numbers of NCBInr database;

^c^Theoretical mass (kDa) and *p*I of identified proteins from the database;

^d^Experimental mass (kDa) and *p*I of identified proteins. Experimental values were calculated with Image Master 2D Platinum software and standard molecular mass marker;

^e^Matched number of peptides (MP) identified from PFF data;

^f^The amino acid sequence coverage (SC) for the identified proteins;

^g^The protein location (Loc) predicted by Target P. (S: secretory pathway);

^h^The Mascot searched score (MS) against the database NCBInr.

### Comparative analysis of *SIX1* gene expression in mycelia, conidia and infected cabbage roots

**Fig 3:**
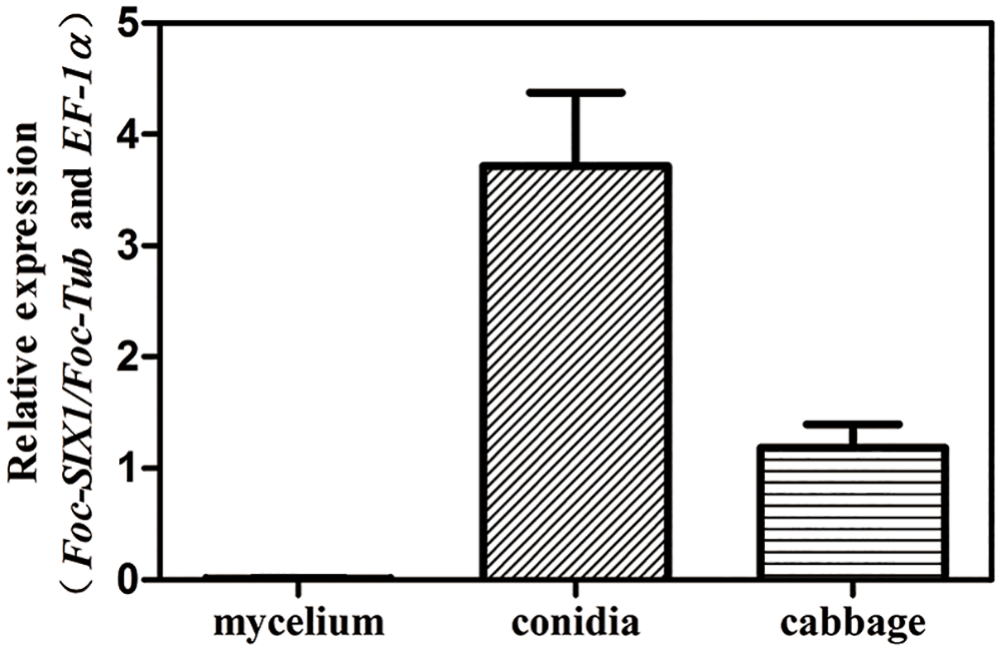
The expression of *SIX1* gene in mycelia, conidia and infected cabbage roots at 7 dpi. Mycelia and conidia were obtained from Foc grown in PDB for 48 h. Gene expression levels were relative to the internal controls *EF-1α* and *β-tubulin*. Averages of four biological replicates were shown with the standard error.

To determine whether *Foc-SIX1* is expressed during infection of cabbage, we quantified its mRNA expression using qRT-PCR in mycelia under axenic culture condition and in infected cabbages. The expression of *SIX1* in mycelia was significantly lower than that in infected cabbage. Its expression was detected in cabbage roots at 90-fold higher levels than when measured in mycelia (Fig 3). These expression levels indicated that dramatically altered expression of *SIX1* could be detected in Foc after cabbage infection. Previous research reported that many effector genes encoding small secreted proteins were low or undetectable under axenic culture conditions and were up-regulated specifically during infection [10]. Van Der Does indicated that *SIX1* gene expression in Fol was much higher *in planta* compared to expression in axenic cultures [9]. This situation also occurred in Foc: *SIX1* in Foc was weakly expressed in mycelia in axenic culture and up-regulated in *planta*, which were consistent with previous investigations. To verify the results based on proteomic analysis, the expression of *SIX1* in conidia was also quantified. As expected, *SIX1* was highly expressed in conidia under axenic culture condition (Fig 3). That was novel compared to the previous researches. The previous researches mainly focused on the expression of *SIX* gene in mycelia under axenic culture and *in planta* conditions.

### Deletion of *SIX1* gene in Foc

**Fig 4:**
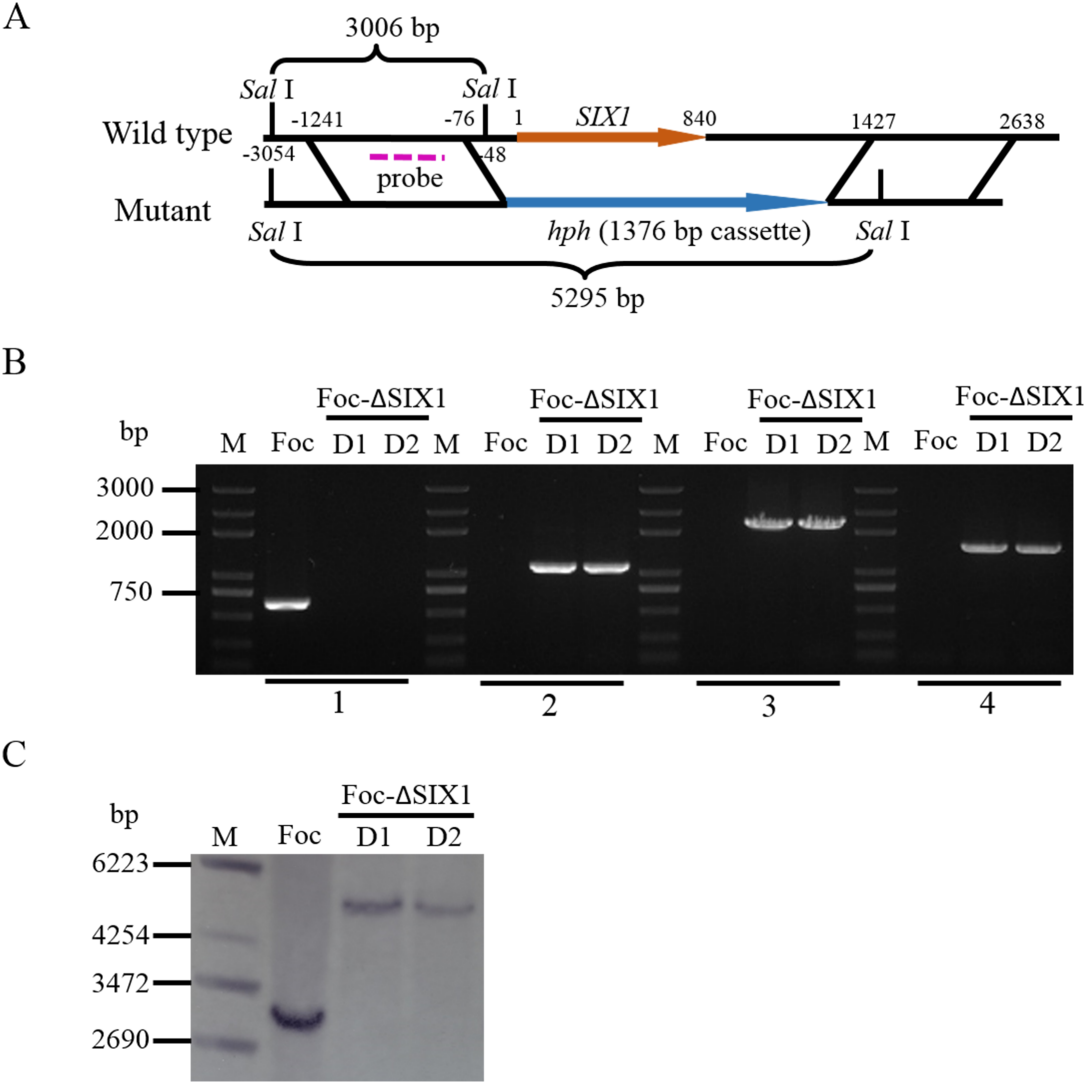
Construction and verification of deletion mutants Foc-ΔSIX1. A: *Foc-SIX1* gene deletion and replacement with an intact selectable marker gene (*hph*) by homologous recombination. B: Confirmation of the correct deletion by PCR. Numbers 1, 2, 3 and 4 represent the four primer pairs that specify the *SIX1* gene, *hph* gene, and the correct upstream and downstream homologous recombination, respectively. C: Southern hybridization analysis of wild type isolate and mutants Foc-ΔSIX1. The genomic DNAs from the wild type Foc isolate and deletion mutants Foc-ΔSIX1 were digested with *Sal* I. A fragment amplified from upstream of the target gene was used as probe.

The deletion mutants Foc-ΔSIX1 were obtained by a targeted gene replacement through homologous recombination. The upstream and downstream regions of the target gene and the selectable marker gene were amplified as shown in S2 Fig (A, B, C, and D). The two split marker transformation components E and F in S2 Fig were obtained by fusion PCR with A and C, B and D. Each transformation component was flanked by a target gene and contained two-thirds of a selectable marker gene. Homologous recombination between the overlapping regions of the selectable marker gene and between the flank regions and their genome counterparts resulted in a target gene deletion and replacement with an intact selectable marker gene (Fig 4A). The mutants Foc-ΔSIX1 were analyzed by PCR to confirm the correct deletion (Fig 4B). All mutants showed that the *SIX1* gene was deleted and that the *hph* gene was integrated into the correct position within the genome. Furthermore, the mutants were also analyzed by Southern hybridization (Fig 4C). Foc-ΔSIX1 showed a larger band compared with wild type isolate. The deletion mutants lacked the 3006 bp band and instead had a 5295 bp band (Fig 4A and 4C).

### Mutants Foc-ΔSIX1 reduced virulence in *F*. *oxysporum* f. sp. *conglutinans* on cabbage

**Fig 5:**
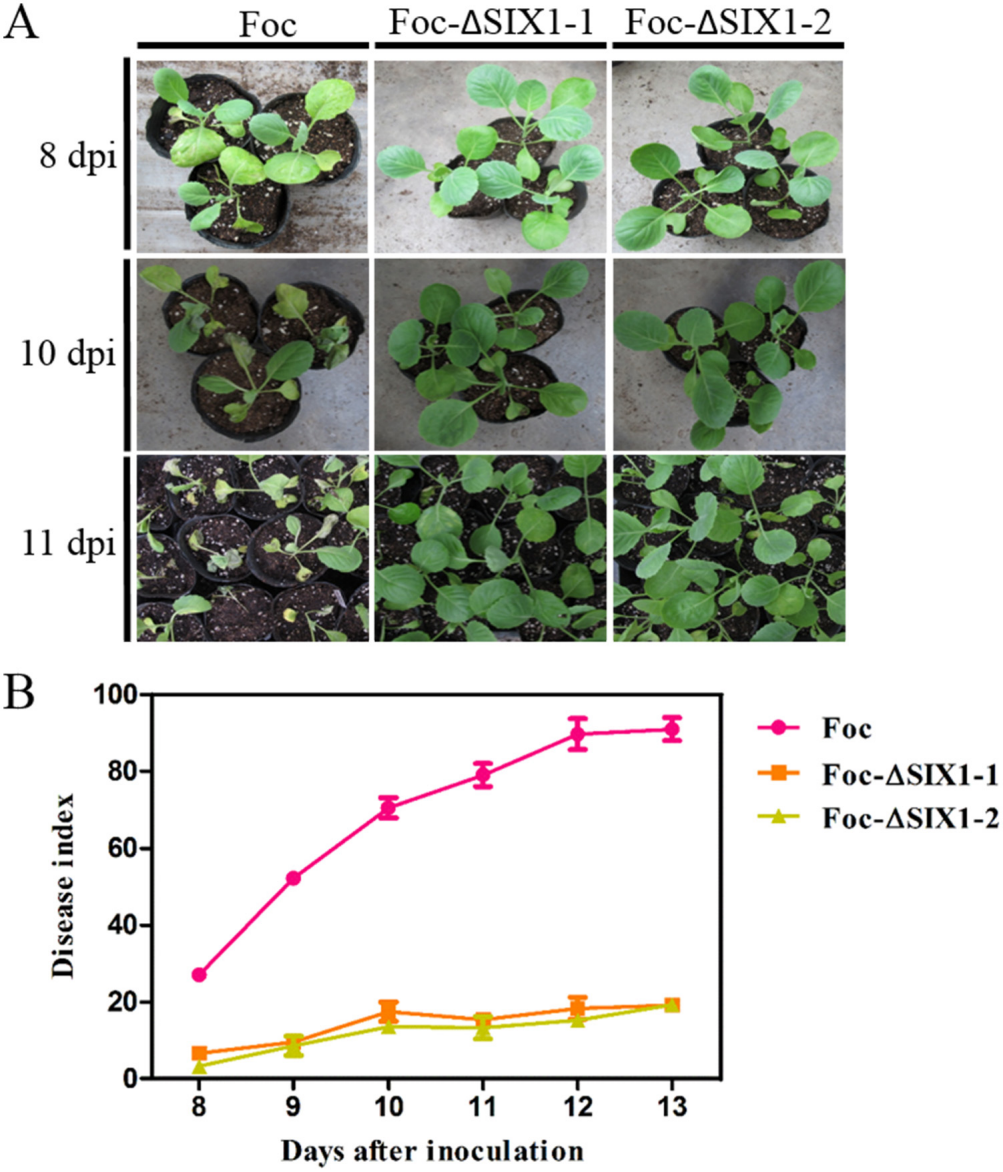
Functional analysis of the *SIX1* gene in Foc. A: Disease symptoms of cabbage seedlings inoculated with wild type isolate Foc and their mutants Foc-ΔSIX1 were shown at 8 dpi, 10 dpi and 11 dpi. B: Disease index of cabbage seedlings inoculated with wild type isolate Foc and mutants Foc-ΔSIX1 from 8 to 13 days after inoculation. The average of three replicates was shown with standard error of the mean. Each value in the figure was an average of three independent biological replicates. Each replicate included more than 30 seedlings for each isolate.

To examine the role of Foc-ΔSIX1 in cabbage infection, we compared the disease symptoms of cabbage when inoculated with wild type isolate and Foc-ΔSIX1 mutants. Foc-ΔSIX1-inoculated cabbage seedlings showed significantly reduced disease symptom development (Fig 5). For example, the yellowing leaves appeared on cabbages inoculated with wild type isolate at 8 dpi, while there were no obvious symptoms on the cabbages inoculated with Foc-ΔSIX1 (Fig 5A). At 10 dpi, severe chlorotic and necrotic symptoms were visualized on the cabbages inoculated with wild type isolate, but the Foc-ΔSIX1-inoculated cabbages still grew well with few visible disease symptoms. Even when the seedlings inoculated with wild type isolate Foc died at 11 dpi, the seedlings inoculated with Foc-ΔSIX1 were still healthy. Note that cabbage seedlings inoculated with wild type isolate Foc not only showed yellowing and wilting but also had limited growth. However, the seedlings inoculated with Foc-ΔSIX1 grew healthily. By comparing the disease indexes between wild type isolate (Foc) and mutants (Foc-ΔSIX1), we concluded that Foc-ΔSIX1 had significantly reduced virulence on cabbage (Fig 5B, S7 Table). The results demonstrated that SIX1 played crucial roles in Foc, which could influence its virulence on cabbage. The growth rates did not differ between wild type isolate (Foc) and mutants (Foc-ΔSIX1) (S3 Fig), suggesting that Foc-SIX1 did not affect fungal growth or development. It was reported that the resistance of cabbage to *Fusarium* wilt was controlled by a single dominant gene *FOC1* [27,28]. SIX1 in Fol acted as an Avr protein where it triggered resistance in I-3-containing tomato [6]. In our work, we also analyzed if *SIX*1 in Foc works as an avirulence gene by the inoculation test using yellow resistance cabbage varieties (“Zhenqi”). The results showed the inoculated seedlings were no disease symptoms even at 15 dpi, whether in Foc-cabbage or Foc-ΔSIX1-cabbage interactions. It indicated that *SIX*1 was not an avirulence gene, but a virulence gene in Foc.

### Restoration of full virulence by complementation of *Foc-SIX1* in the deletion mutants Foc-ΔSIX1

**Fig 6:**
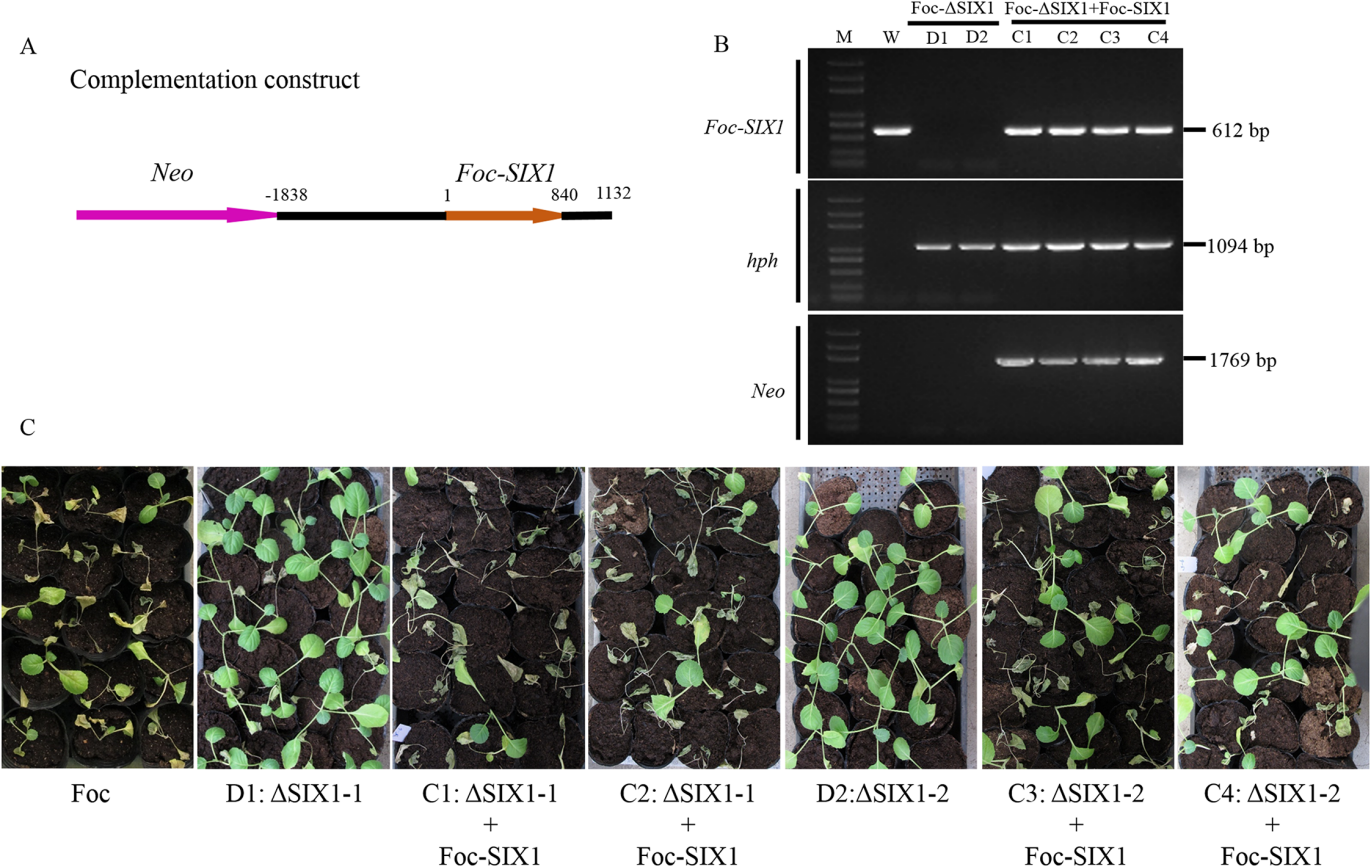
Construction, verification, and virulence analysis of complementation mutants Foc-ΔSIX1::Foc-SIX1. A: Foc-ΔSIX1 mutants were complemented by transformation with approximately 3.0 kb of *Foc-SIX1* spanning from -1838 bp (promoter relative to start codon) to 1132 bp (ORF and 3’ UTR). B: The *SIX1* gene, *hph* gene and *Neo* gene fragments were amplified by PCR to confirm the correct complementation mutants. C: Disease symptoms of cabbage seedlings inoculated with wild type isolate Foc, deletion mutants Foc-ΔSIX1, and complementation mutants Foc-ΔSIX1::Foc-SIX1 were shown at 14 dpi. Similar results were obtained in three independent biological replicates.

To confirm that the reduced virulence of mutants Foc-ΔSIX1 was due to deletion of *SIX1*, deletion mutants Foc-ΔSIX1 were complemented with an intact copy of *Foc-SIX1* fragment (Fig 6A). Complementation of Foc-ΔSIX1 was confirmed by PCR amplification (Fig 6B). Foc-SIX1-complemented isolates (C1-C4: Foc-ΔSIX1::Foc-SIX1) showed that *SIX1* gene was complemented, and *hph* gene and *Neo* gene were integrated into the genome. After inoculation, all of Foc-SIX1-complemented isolates showed full virulence symptom (Fig 6C). The disease indexes for the complemented isolates were not significantly different from that of wild type isolate (Foc) and were significantly higher than that of the two deletion mutants (D1 and D2) (S8 Table). After complementation, Foc-SIX1-complemented isolates restored full Foc virulence on cabbage. It demonstrated that *SIX1* gene was required for full virulence of Foc on cabbage.

### No complementation of Foc-ΔSIX1 by a Fol-SIX1, the Foc-SIX1 homolog in *F*. *oxysporum* f. sp. *lycopersici*

**Fig 7:**
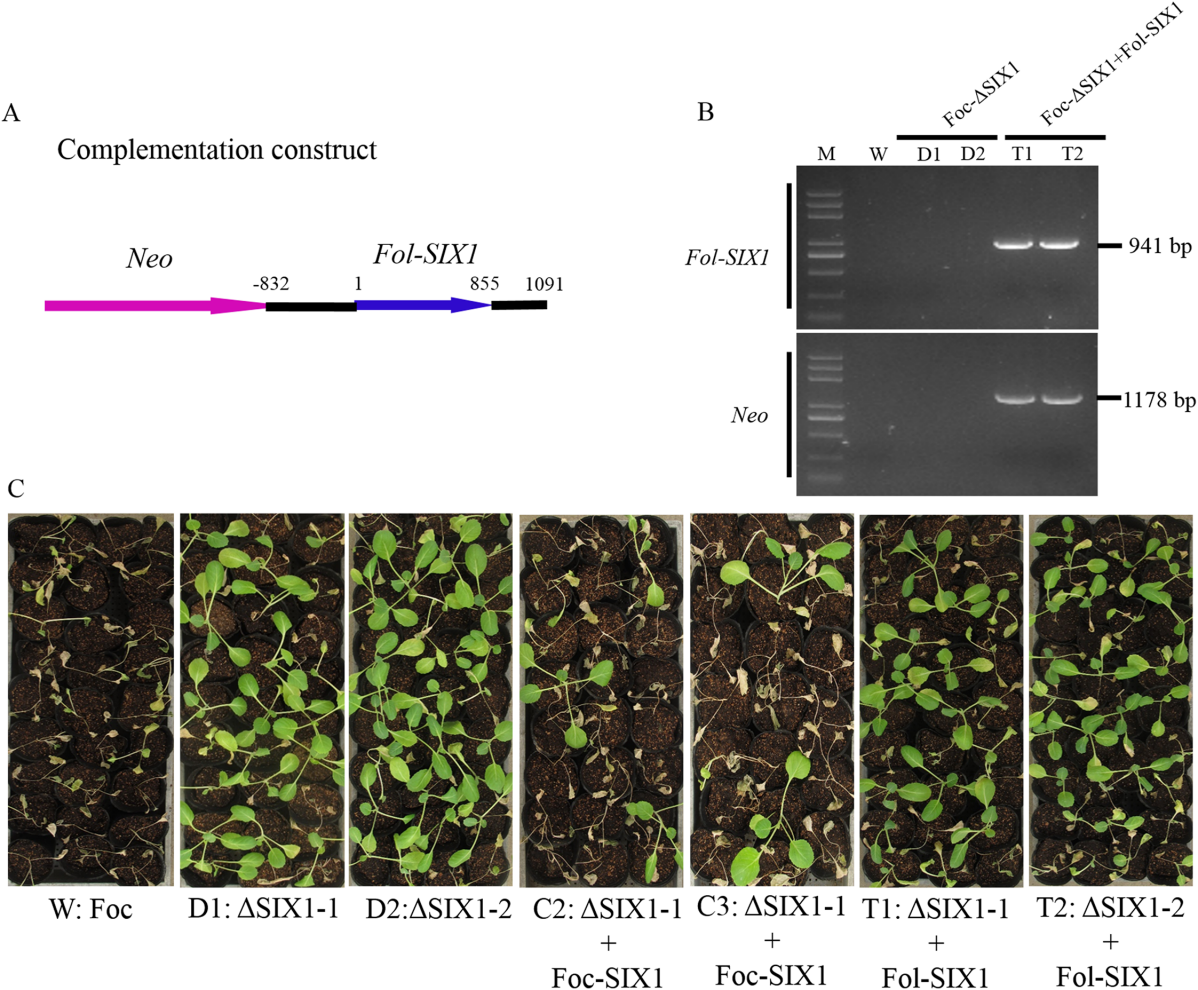
Construction, verification, and virulence analysis of transformants Foc-ΔSIX1::Fol-SIX1. A: Foc-ΔSIX1 mutants were complemented by transformation with approximately 2.0 kb of *Fol-SIX1* spanning from -832 bp (promoter relative to start codon) to 1091 bp (ORF and 3’ UTR). B: The *Fol-SIX1* gene and *Neo* gene fragments were amplified by PCR to confirm the correct complementation mutants. C: Disease symptoms of cabbage seedlings inoculated with wild type isolate Foc, deletion mutants Foc-ΔSIX1 (D1 and D2), complementation mutants (C2 and C3) and transformants Foc-ΔSIX1::Fol-SIX1 (T1 and T2) were shown at 18 dpi. Similar results were obtained in three independent biological replicates.

To test whether SIX1 might have a nonspecific effect on virulence, we also introduced Fol-SIX1, which was a Foc-SIX1 homolog in Fol, into Foc-ΔSIX1 mutants (Fig 7A). Foc-ΔSIX1::Fol-SIX1 was confirmed by PCR amplification (Fig 7B). The results showed no difference in disease symptom development between Foc-ΔSIX1 and Foc-ΔSIX1::Fol-SIX1 strains when infected cabbage (Fig 7C). Disease development caused by both wild type strain Foc and complemented mutants Foc-ΔSIX1::Foc-SIX1 was significantly higher than that caused by the two Foc-ΔSIX1 mutants and the two Foc-ΔSIX1::Fol-SIX1 mutants. The disease indexes for complemented isolates (T1 and T2: transformants Foc-ΔSIX1::Fol-SIX1) were not significantly different from those of Foc-ΔSIX1 mutants and were significantly lower than that of wild type isolate (Foc) and the two complemented mutants (C2 and C3: Foc-ΔSIX1::Foc-SIX1) (S9 Table). These experiments confirmed that Fol-SIX1 was not capable of replacing the role of Foc-SIX1 in Foc on the disease symptom development of cabbage. It had been reported that Fo5176-SIX4 and Fol-SIX4 could restore the virulence of Fo5176-ΔSIX4. The expression of Fo5176-SIX4 in *A*. *thaliana* increased susceptibility to Fo5176 [11]. While Fol-SIX6 did not promote growth of *A*. *tumefaciens* in *N*. *benthamiana* [29]. The Fol-SIX6 expression in *A*. *thaliana* could not enhance susceptibility to Fo5176 or *V*. *dahliae* [29]. Our research confirmed that Fol-SIX1 did not alter the virulence of Foc-ΔSIX1 isolate on cabbage. The roles of Fol-SIX1 on virulence might rely on the host specificity. The roles of Foc-SIX1 has led us to study its molecular mechanism in further detail. Further research is required to determine the roles played by SIX1 in the conidia of Foc and to notably decrease Foc virulence on cabbage.

## Supporting Information

S1 Fig Overlap PCR and split marker gene disruption strategy. A. Amplification of 5’- and 3’- flanking regions of the target gene and split replacement of selectable marker gene. B. Fusion the flanking regions and split marker sequences. C. Homologous recombination between the flank regions of the target gene and their genome counterparts and between the overlapping regions of the selectable marker gene.
(DOCX)

S2 Fig The construction of transformation component. A and B. The upstream and downstream homologous recombination fragments of target gene. C and D. The split replacements of *hph* gene. E and F. The fusion fragments between A and C, B and D, which were directly used for protoplast-mediated transformation. M. Marker.
(DOCX)

S3 Fig The analysis of fungal growth rates between wild type isolate Foc and deletion mutants Foc-ΔSIX1. (DOCX)

S1 File Spectrum and MALDI TOF/TOF MS/MS identification information for the identified protein Foc-SIX1. (DOCX)

S1 Table The primer pairs used in qRT-PCR analysis. (DOCX)

S2 Table The primer pairs for deletion construction of *SIX1* gene. (DOCX)

S3 Table The primer pairs used to ensure the correct deletion mutants. (DOCX)

S4 Table The primer pairs used for complementation *Foc-SIX1* in Foc-ΔSIX1. (DOCX)

S5 Table The primer pairs used for complementation homolog *Fol-SIX1* in Foc-ΔSIX1. (DOCX)

S6 Table Detailed information on MS identification, functional classification and GO analysis of the four protein spots. (XLSX)

S7 Table Disease index on cabbage seedlings inoculated with wild type isolate Foc and mutants Foc-ΔSIX1 at 8, 10 and 12 dpi. (DOCX)

S8 Table Disease index on cabbage seedlings inoculated with wild type Foc, deletion mutants Foc-ΔSIX1 (D1 and D2) and complementation mutants Foc-ΔSIX1::Foc-SIX1 (C1, C2, C3 and C4) at 14 dpi. (DOCX)

S9 Table Disease index on cabbage seedlings inoculated with wild type Foc, Foc-ΔSIX1 (D1 and D2), Foc-ΔSIX1::Foc-SIX1 (C2 and C3) and Foc-ΔSIX1::Fol-SIX1 (T1 and T2) at 18 dpi. (DOCX)
