# Supplementary material for: A SIX1 Homolog in Fusarium oxysporum f. sp. conglutinans Is Required for Full Virulence on Cabbage
Source: PLoS One. 2016 Mar 24;11(3):e0152273. doi: 10.1371/journal.pone.0152273 (PMC4807099; doi:10.1371/journal.pone.0152273)
Supplement: S1 Fig — A. Amplification of 5’- and 3’- flanking regions of the target gene and split replacement of selectable marker gene. B. Fusion the flanking regions and split marker sequences. C. Homologous recombination between the flank regions of the target gene and their genome counterparts and between the overlapping regions of the selectable marker gene. (DOCX) [file pone.0152273.s001.docx]

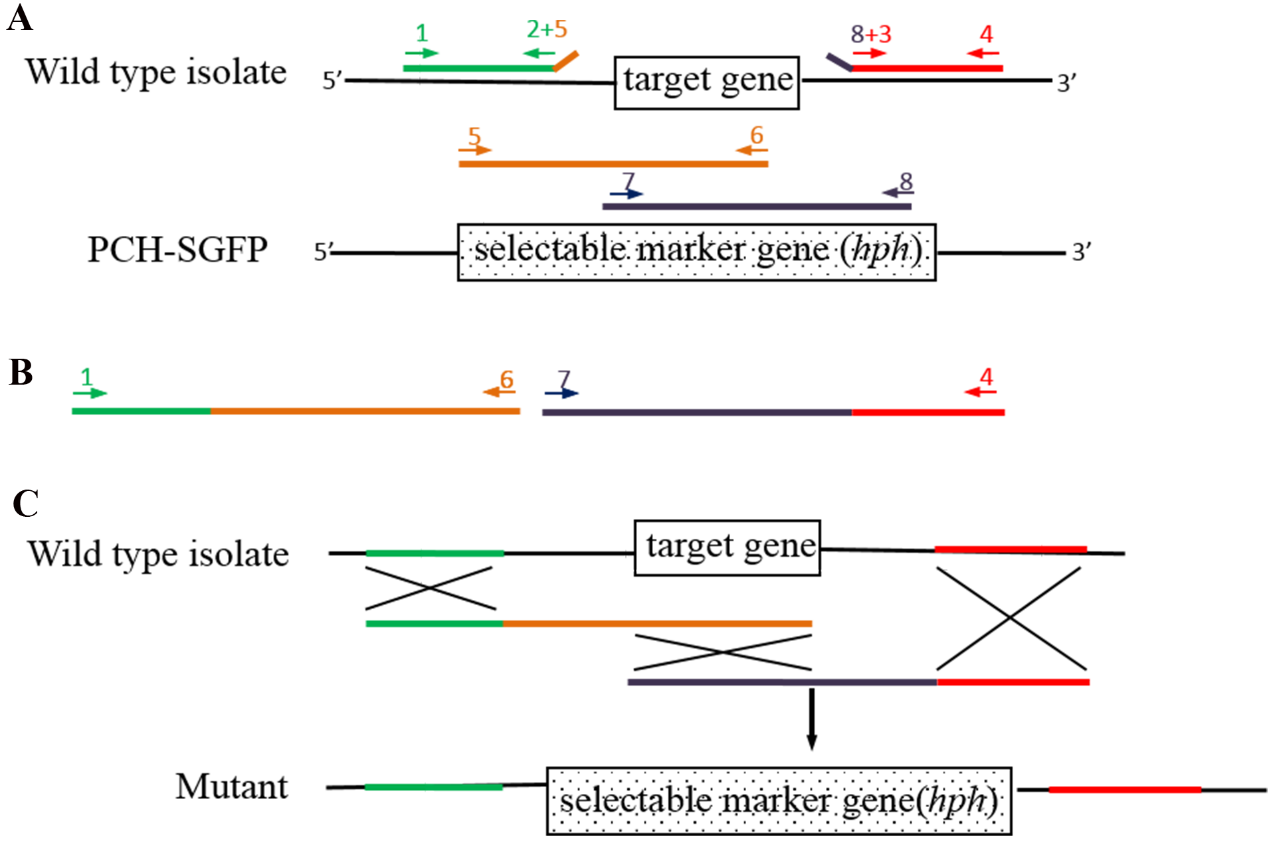


**S1 Fig. Overlap PCR and split marker gene disruption strategy.** A. Amplification of 5’- and 3’- flanking regions of the target gene and split replacement of selectable marker gene. B. Fusion the flanking regions and split marker sequences. C. Homologous recombination between the flank regions of the target gene and their genome counterparts and between the overlapping regions of the selectable marker gene.
