# Supplementary material for: A SIX1 Homolog in Fusarium oxysporum f. sp. conglutinans Is Required for Full Virulence on Cabbage
Source: PLoS One. 2016 Mar 24;11(3):e0152273. doi: 10.1371/journal.pone.0152273 (PMC4807099; doi:10.1371/journal.pone.0152273)
Supplement: S2 Fig — A and B. The upstream and downstream homologous recombination fragments of target gene. C and D. The split replacements of hph gene. E and F. The fusion fragments between A and C, B and D, which were directly used for protoplast-mediated transformation. M. Marker. (DOCX) [file pone.0152273.s002.docx]

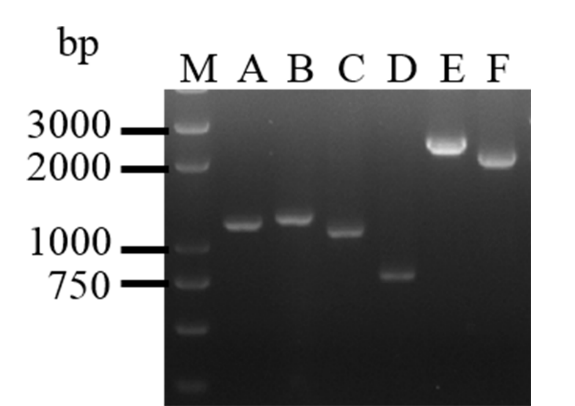


**S2 Fig. The construction of transformation component.** A and B. The upstream and downstream homologous recombination fragments of target gene. C and D. The split replacements of *hph* gene. E and F. The fusion fragments between A and C, B and D, which were directly used for protoplast-mediated transformation. M. Marker.
