# Supplementary material for: A SIX1 Homolog in Fusarium oxysporum f. sp. conglutinans Is Required for Full Virulence on Cabbage
Source: PLoS One. 2016 Mar 24;11(3):e0152273. doi: 10.1371/journal.pone.0152273 (PMC4807099; doi:10.1371/journal.pone.0152273)
Supplement: S3 Fig — (DOCX) [file pone.0152273.s003.docx]

**S3 Fig. The analysis of fungal growth rates between wild type isolate Foc and deletion mutants Foc-∆SIX1.**
