# Supplementary material for: A SIX1 Homolog in Fusarium oxysporum f. sp. conglutinans Is Required for Full Virulence on Cabbage
Source: PLoS One. 2016 Mar 24;11(3):e0152273. doi: 10.1371/journal.pone.0152273 (PMC4807099; doi:10.1371/journal.pone.0152273)

**Supplemental spectra and MALDI TOF/TOF MS/MS identification information for Foc-SIX1**

**MALDI TOF:**

matrix assisted laser desorption ionization time of flight

**MS:** mass spectrometry

**PFF:** peptide fragment fingerprinting

Spot No.: **1**

NCBI accession No.: **gi|342888423**

Species: ***Fusarium oxysporum* Fo5176**

Protein name: **Fo5176-SIX1**

PFF score: **[86]**

Matched peptides No.: [1] Sequence coverage %: [5] Matched sequences: **R.GNGHSWACINCPGGK.L**

Calculated Mr: **31302** Calculated *p*I: **8.54**

Probability Based Mowse Score:


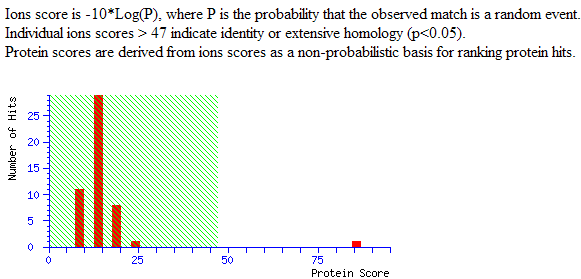


Matched peptide sequences: shown in Bold Red:


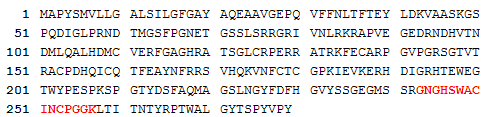


Spot No.: **2**

NCBI accession No.: **gi|342888423**

Species: ***Fusarium oxysporum* Fo5176**

Protein name: **Fo5176-SIX1**

PFF score: **[58]**

Matched peptides No.: **[1]**  Sequence coverage %: **[5]** Matched sequences: **R.GNGHSWACINCPGGK.L**

Calculated Mr: **31302** Calculated *p*I: **8.54**

Probability Based Mowse Score:


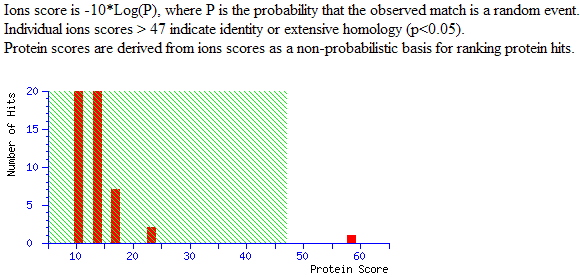


Matched peptide sequences: shown in Bold Red:


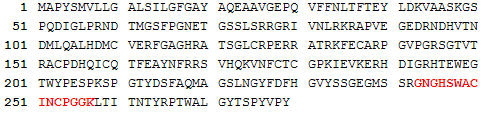


Spot No.: **3**

NCBI accession No.: **gi|342888423**

Species: ***Fusarium oxysporum* Fo5176**

Protein name: **Fo5176-SIX1**

PFF score: **[182]**

Matched peptides No.: **[2]**  Sequence coverage %: **[10]** Matched sequences: **R.HTEWEGTWYPESPK.S**

**R.GNGHSWACINCPGGK.L**

Calculated Mr: **31302** Calculated *p*I: **8.54**

Probability Based Mowse Score:


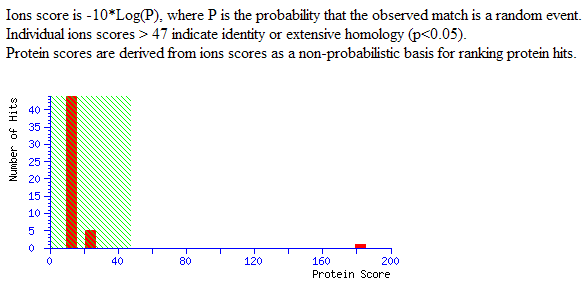


Matched peptide sequences: shown in Bold Red:


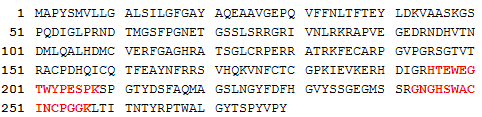


Spot No.:**4**

NCBI accession No.: **gi|342888423**

Species: ***Fusarium oxysporum* Fo5176**

Protein name: **Fo5176-SIX1**

PFF score: **[65]**

Matched peptides No.: **[1]** Sequence coverage %: **[5]** Matched sequences: **R.HTEWEGTWYPESPK.S**

Calculated Mr: **31302**  Calculated *p*I: **8.54**

Probability Based Mowse Score:


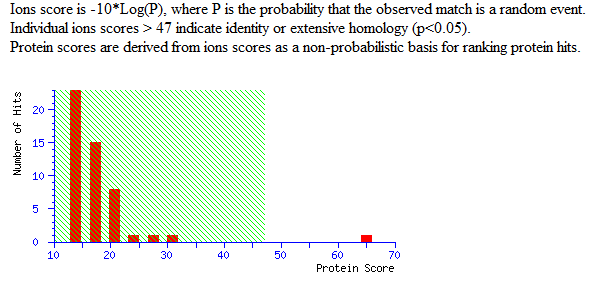


Matched peptide sequences: shown in Bold Red:


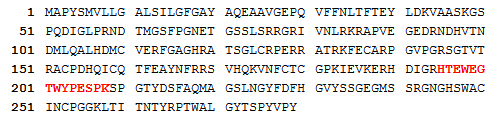

Supplement: S1 File — (DOCX) [file pone.0152273.s004.docx]
