# Supplementary material for: A SIX1 Homolog in Fusarium oxysporum f. sp. conglutinans Is Required for Full Virulence on Cabbage
Source: PLoS One. 2016 Mar 24;11(3):e0152273. doi: 10.1371/journal.pone.0152273 (PMC4807099; doi:10.1371/journal.pone.0152273)
Supplement: S1 Table — (DOCX) [file pone.0152273.s005.docx]

**S1 Table. The primer pairs used in qRT-PCR analysis.**

| Gene | Accession No. | Protein name | Sequences |
| --- | --- | --- | --- |
| *Foc-SIX1* | 342888423 | Fo5176-SIX1 | 5'-TCAAGAGGCTGCGGTTGG-3'  5'-GACGCTCAGGGCGACATA-3' |
| *Foc-Tub* | FOXG_06228 | Fo-β-tubulin | 5'-TGTTCGACCCCAAGAACAT-3'  5'-GGTCCTCGACCTCCTTCATA-3' |
| *Foc-EF* | 112820563 | Fo-EF-1α | 5'-CATCGGCCACGTCGACTCT-3'  5'-AGAACCCAGGCGTACTTGAA-3' |
