# Supplementary material for: A SIX1 Homolog in Fusarium oxysporum f. sp. conglutinans Is Required for Full Virulence on Cabbage
Source: PLoS One. 2016 Mar 24;11(3):e0152273. doi: 10.1371/journal.pone.0152273 (PMC4807099; doi:10.1371/journal.pone.0152273)
Supplement: S2 Table — (DOCX) [file pone.0152273.s006.docx]

**S2 Table. The primer pairs for deletion construction of *SIX1* gene.**

| Gene | Fragment ^a^ | | Primers | Sequences |
| --- | --- | --- | --- | --- |
| Foc-SIX1 | A | 1-F  (2+5)-R | | 5’-TGTAAGGGATCAGGGTGC -3’  5’-ACCTCCACTAGCTCCAGCCAAGTGGCTCGTGAGACAAAGTAA-3’ |
|  | B | (8+3)-F  4-R | | 5’-GAATAGAGTAGATGCCGACCGGGGCCAGGTTTGAGATGACG-3’  5’-GGTGAGGACTTGCGACTT-3’ |
|  | C | 5-F  6-R | | 5’-CTTGGCTGGAGCTAGTGGAGGT-3’  5’-GGATGCCTCCGCTCGAAGTA-3’ |
|  | D | 7-F  8-R | | 5’-CGTTGCAAGACCTGCCTGAA-3’  5’-CCCGGTCGGCATCTACTCTATTC-3’ |
|  |  | Probe-F  Probe-R | | 5’-TCGGCACCTGAATGAAAC-3’  5’-TGGCTCGTGAGACAAAGTAA-3’ |

1. The corresponding amplified fragments were marked in the S2 Fig .
