# Supplementary material for: A SIX1 Homolog in Fusarium oxysporum f. sp. conglutinans Is Required for Full Virulence on Cabbage
Source: PLoS One. 2016 Mar 24;11(3):e0152273. doi: 10.1371/journal.pone.0152273 (PMC4807099; doi:10.1371/journal.pone.0152273)
Supplement: S3 Table — (DOCX) [file pone.0152273.s007.docx]

**S3 Table. The primer pairs used to ensure the correct deletion mutants.**

| Fragment ^a^ | Primers | Sequences |  |
| --- | --- | --- | --- |
| 1 | 1-F  1-R | 5’-CGCATCGTCAACCTTAGAA-3’  5’-CGCAACTTAGTAGGGGACAT-3’ | |
| 2 | 2-F  2-R | 5’-CTTGGCTGGAGCTAGTGGAGGT-3’  5’-GGATGCCTCCGCTCGAAGTA-3’ | |
| 3 | 3-F  3-R | 5’-GGGTGACGGACGTTAGTT-3’  5’-GGATGCCTCCGCTCGAAGTA-3’ | |
| 4 | 4-F  4-R | 5’-TCTGGACCGATGGCTGTGTAG-3’  5’-ATGACAGCCGATGGGATA-3’ | |

1. The corresponding amplified fragments were marked in Fig 3B.
