# Supplementary material for: A SIX1 Homolog in Fusarium oxysporum f. sp. conglutinans Is Required for Full Virulence on Cabbage
Source: PLoS One. 2016 Mar 24;11(3):e0152273. doi: 10.1371/journal.pone.0152273 (PMC4807099; doi:10.1371/journal.pone.0152273)
Supplement: S4 Table — (DOCX) [file pone.0152273.s008.docx]

**S4 Table. The primer pairs used for complementation *Foc-SIX1* in Foc-∆SIX1.**

| Primers | Sequences | |
| --- | --- | --- |
| Foc-SIX1-F (*Bam*HI)  Foc-SIX1-R (*Eco*RI) | | 5’-CGCGGATCCGCGACAGATGAGCGAGACAATG-3’  5’-CCGGAATTCCGGCGAATACGATACCTACGG-3’ |
| Foc-SIX1-F  Foc-SIX1-R | | 5’-CGCATCGTCAACCTTAGAA-3’  5’-CGCAACTTAGTAGGGGACAT-3’ |
| hph-F  hph-R | | 5’-CTTGGCTGGAGCTAGTGGAGGT-3’  5’-GGATGCCTCCGCTCGAAGTA-3’ |
| Neo-F  Neo-R | | 5’-TAGGGCGAATTGGGTAC-3’  5’-CAGCTCACTGTTCACGTC-3’ |
