# Supplementary material for: A SIX1 Homolog in Fusarium oxysporum f. sp. conglutinans Is Required for Full Virulence on Cabbage
Source: PLoS One. 2016 Mar 24;11(3):e0152273. doi: 10.1371/journal.pone.0152273 (PMC4807099; doi:10.1371/journal.pone.0152273)
Supplement: S5 Table — (DOCX) [file pone.0152273.s009.docx]

**S5 Table. The primer pairs used for complementation *Fol_SIX1* homolog in Foc-∆SIX1.**

| Primers | Sequences | |
| --- | --- | --- |
| Fol-SIX1-F (*Bam*HI)  Fol-SIX1-R (*Eco*RI) | | 5’-CGGGATCCCGAGTGATGCTCGATACAACCCTC-3’  5’-GGAATTCCGTTGGAGGTCGCGTTCTTGTAC-3’ |
| Fol-SIX1-F  Fol-SIX1-R | | 5’-GCCTGCCCGATTCTAAAC-3’  5’-ACACCGTCATACCCGAAG-3’ |
| Neo-F  Neo-R | | 5’-ACCAACCTGGAAACACCTC-3’  5’-CACTGTAACCCGCAACGAA-3’ |
