# Supplementary material for: A SIX1 Homolog in Fusarium oxysporum f. sp. conglutinans Is Required for Full Virulence on Cabbage
Source: PLoS One. 2016 Mar 24;11(3):e0152273. doi: 10.1371/journal.pone.0152273 (PMC4807099; doi:10.1371/journal.pone.0152273)
Supplement: S7 Table — (DOCX) [file pone.0152273.s011.docx]

**S7 Table. Disease index on cabbage seedlings inoculated with wild type isolate Foc and mutants Foc-∆SIX1 at 8, 10 and 12 dpi.**

| **Isolate** | **Disease index (DI)** | | |
| --- | --- | --- | --- |
|  | **8 dpi** | **10 dpi** | **12 dpi** |
| **Foc (52557^-TM^)**  **Foc-∆SIX1-1**  **Foc-∆SIX1-2** | 27.03±1.84A | 70.47±2.63A | 89.63±4.05A |
|  | 6.67±1.44B | 17.50±2.50C | 18.33±2.89C |
|  | 3.23±1.54B | 13.53±1.79C | 15.20±0.35C |
| **Mock (H_2_O)** | 0.00±0.00 | 0.00±0.00 | 0.00±0.00 |

The values within columns followed by different letters were all significantly different from each other according to Duncan's multiple range test at *P*<0.01. Each value in the table was an average of three independent biological replicates with standard errors of the mean. Each replicate included more than 30 seedlings for each isolate.
