# Supplementary material for: A SIX1 Homolog in Fusarium oxysporum f. sp. conglutinans Is Required for Full Virulence on Cabbage
Source: PLoS One. 2016 Mar 24;11(3):e0152273. doi: 10.1371/journal.pone.0152273 (PMC4807099; doi:10.1371/journal.pone.0152273)
Supplement: S8 Table — (DOCX) [file pone.0152273.s012.docx]

**S8 Table. Disease index on cabbage seedlings inoculated with wild type Foc, deletion mutants Foc-∆SIX1 (D1 and D2) and complementation mutants Foc-∆SIX1::Foc-SIX1 (C1, C2, C3 and C4) at 14 dpi.**

| **Isolate** | **Disease index (DI)** |
| --- | --- |
|  | **14 dpi** |
| **Foc (52557^-TM^)**  **D1: Foc-∆SIX1-1**  **C1: Foc-∆SIX1-1::Foc-SIX1** | 91.28±5.35A |
|  | 27.11±2.47B |
|  | 96.67±1.76A |
| **C2: Foc-∆SIX1-1::Foc-SIX1** | 94.48±1.76A |
| **D2: Foc-∆SIX1-2** | 21.67±2.34B |
| **C3: Foc-∆SIX1-2::Foc-SIX1** | 83.96±0.88A |
| **C4: Foc-∆SIX1-2::Foc-SIX1** | 89.09±5.15A |
| **Mock (H_2_O)** | 0.00±0.00 |

The values within columns followed by different letters were significantly different from each other according to Duncan's multiple range test at *P*<0.01. Each value in the table was an average of three independent biological replicates with standard errors of the mean.
