# Supplementary material for: A SIX1 Homolog in Fusarium oxysporum f. sp. conglutinans Is Required for Full Virulence on Cabbage
Source: PLoS One. 2016 Mar 24;11(3):e0152273. doi: 10.1371/journal.pone.0152273 (PMC4807099; doi:10.1371/journal.pone.0152273)
Supplement: S9 Table — (DOCX) [file pone.0152273.s013.docx]

**S9 Table. The primer pairs used for complementation *Fol_SIX1* homolog in Foc-∆SIX1.**

| **Isolate** | **Disease index (DI)** |
| --- | --- |
|  | **18 dpi** |
| **Foc (52557^-TM^)**  **D1: Foc-∆SIX1-1**  **D2: Foc-∆SIX1-2** | 100±0.00A |
|  | 72.22±3.33B |
|  | 68.33±3.00B |
| **C2: Foc-∆SIX1-1::Foc-SIX1** | 95.14±2.54A |
| **C3: Foc-∆SIX1-1::Foc-SIX1** | 94.64±2.70A |
| **T1: Foc-∆SIX1-2::Foc-SIX1** | 75.43±0.57B |
| **T2: Foc-∆SIX1-2::Foc-SIX1** | 73.85±1.03B |
| **Mock (H_2_O)** | 0.00±0.00 |

The values within columns followed by different letters were significantly different from each other according to Duncan's multiple range test at *P*<0.01. Each value in the table was an average of three independent biological replicates with standard errors of the mean.
